# Sporotrichosis: An Overview and Therapeutic Options

**DOI:** 10.1155/2014/272376

**Published:** 2014-12-29

**Authors:** Vikram K. Mahajan

**Affiliations:** Department of Dermatology, Venereology & Leprosy, Dr. R. P. Govt. Medical College, Kangra, Tanda, Himachal Pradesh 176001, India

## Abstract

Sporotrichosis is a chronic granulomatous mycotic infection caused by *Sporothrix schenckii*, a common saprophyte of soil, decaying wood, hay, and sphagnum moss, that is endemic in tropical/subtropical areas. The recent phylogenetic studies have delineated the geographic distribution of multiple distinct *Sporothrix* species causing sporotrichosis. It characteristically involves the skin and subcutaneous tissue following traumatic inoculation of the pathogen. After a variable incubation period, progressively enlarging papulo-nodule at the inoculation site develops that may ulcerate (fixed cutaneous sporotrichosis) or multiple nodules appear proximally along lymphatics (lymphocutaneous sporotrichosis). Osteoarticular sporotrichosis or primary pulmonary sporotrichosis are rare and occur from direct inoculation or inhalation of conidia, respectively. Disseminated cutaneous sporotrichosis or involvement of multiple visceral organs, particularly the central nervous system, occurs most commonly in persons with immunosuppression. Saturated solution of potassium iodide remains a first line treatment choice for uncomplicated cutaneous sporotrichosis in resource poor countries but itraconazole is currently used/recommended for the treatment of all forms of sporotrichosis. Terbinafine has been observed to be effective in the treatment of cutaneous sporotrichosis. Amphotericin B is used initially for the treatment of severe, systemic disease, during pregnancy and in immunosuppressed patients until recovery, then followed by itraconazole for the rest of the therapy.

## 1. Introduction

Deep mycoses involving the skin and/or subcutaneous tissue (subcutaneous mycoses), fascial planes and bones, and/or various organs systems (deep mycoses) account for almost 1% of the total mycoses cases. In most instances of subcutaneous mycoses, infection occurs following traumatic implantation of the etiologic fungi that are saprophytes to the soil and plant detritus. Although once considered endemic in tropical countries, these opportunistic infections are being increasingly observed across populations following accidental exposure to pathogen especially among returning travelers/workers. Current era of immunosuppression due to HIV infection, immunosuppressive therapy for cancers, autoimmune diseases, or organ transplantation has further contributed towards their increased prevalence. While chromoblastomycosis and phaeohyphomycosis, mycetomas, subcutaneous zygomycosis (entomophthoromycosis and mucormycosis), hyalohyphomycosis, and lobomycosis have limited area-specific presence, sporotrichosis, a subcutaneous mycotic infection from* Sporothrix schenckii *species complex, perhaps remains the most reported subcutaneous mycosis worldwide. The heterogeneous morphology of lesions (nodules, plaques, noduloulcerative, ulcerative, nodulocystic or warty lesions, discharging sinuses, and subcutaneous swellings or masses) often makes the clinical diagnosis difficult particularly in nonendemic areas leading to delayed treatment and protracted clinical course causing significant morbidity and impact on public health. In majority, treatment becomes imperative as spontaneous resolution occurs as an exception [1]. Extracutaneous sporotrichosis is also an emerging mycosis in HIV infected patients [2, 3]. This paper presents an overview of sporotrichosis and therapeutic options.

## 2. Epidemiology

This chronic granulomatous subcutaneous mycotic infection is caused by* Sporothrix schenckii *species complex, a common saprophyte of soil, decaying wood, hay, and sphagnum moss. Recent molecular studies have demonstrated that* S. schenckii* is a complex of at least six clinicoepidemiologically important species with significant differences in geographical distribution, biochemical properties (dextrose, sucrose, and raffinose assimilation), degree of virulence, different disease patterns, and response to therapy. These include* S. albicans*,* S. brasiliensis *(in Brazil),* S. mexicana *(in Mexico),* S. globosa* (in UK, Spain, Italy, China, Japan, USA, and India), and* S. schenckii* sensu stricto [4–9]. Hence, the nomenclature “*Sporothrix schenckii *species complex” is preferred to earlier “*Sporothrix schenckii*” that was used to describe the strains from all over the world. According to Marimon et al. [7] human infections are mainly associated with* S. schenckii* sensu stricto,* Sporothrix brasiliensis,* and* Sporothrix globosa* while* Sporothrix mexicana* have only been identified among isolates of environmental origin with occasional exception [8, 10]. Henceforth, the nomenclature “*Sporothrix schenckii*” is used to represent the “*Sporothrix schenckii *species complex.”

Sporotrichosis occurs worldwide with focal areas of hyperendemicity. It is particularly common in tropical/subtropical areas and temperate zones with warm and humid climate favoring the growth of saprophytic fungus but large outbreaks have occurred in other parts as well [11, 12]. Its worldwide incidence is unknown but Japan, China, Australia, Central and South America (Mexico, Brazil, Colombia, and Peru), and India (along the Sub-Himalayan region) account for most frequent occurrences [13–16]. Approximately, one case occur per 1000 people in Peru and in US 200–250 cases (1-2 cases per million) occur annually.

No age, gender, or race is spared of this infection as its occurrence depends upon the fungus in the environment and the portal of entry. The preponderance of males in most reported cases is attributed to their higher exposure risk than gender susceptibility. The traumatic inoculation is the obvious reason that exposed body parts, the extremities in particular, are involved most frequently; the upper limbs are affected twice as commonly as the lower limbs and involvement is infrequent [14, 17, 18].

The disease is almost endemic in rural areas and professionals handling plants or plant material such as farmers, gardeners, florists, foresters, and nursery workers are particularly at higher risk. The majority of these patients are between 20 and 50 years of age; the most active years of life when the individual is probably exposed maximally to injuries [14].* S. schenckii* gains entry into the skin by traumatic implantation from contaminated thorns, hay stalks, barbs, soil, splinters, and bizarre/roadside injuries leading to cutaneous infection [14]. However, only 10–62% of patients recall any history of trauma as it is usually innocuous, occurs few weeks earlier, and is mostly forgotten [1, 14]. Although animals are not significant source of infection in humans, zoonotic transmission has been reported from insect bites, fish handling, and bites of cats, birds, dogs, rats, reptiles, and horses [19, 20]. Cats have been found to be important vehicle in dissemination of* S. schenckii* in a long-lasting epidemic of sporotrichosis in Brazil diagnosed by isolation of* S. schenckii* (*S. brasiliensis* in 97% isolates) from different types of samples, both from humans and from feline, indicating the emergence of another set of at-risk personnel [20–24]. Human-to-human spread, mostly from wound contamination from infected dressings or indigenous/herbal topical medication, interestingly remains underestimated [14, 25].

## 3. Clinical Presentations

Its exact incubation period remains unknown and may range from a few days to a few months, the average being 3 weeks [26]. The skin and the surrounding lymphatics are involved primarily leading to development of a small, indurated, progressively enlarging papulo-nodule at the inoculation site that may ulcerate (sporotrichotic chancre) without causing systemic symptoms. It may remain as such, or develops multiple lesions. Thereafter, sporotrichosis is presented in three main clinical types: lymphocutaneous, fixed cutaneous, and multifocal or disseminated cutaneous sporotrichosis. Extracutaneous or systemic sporotrichosis occurs from hematogenous spread from the primary inoculation site, the lymph node, or more usually from pulmonary disease in immunosuppressed patients. In children clinical profile is almost similar but facial involvement is more frequent accounting for 40–60% or as high 97% in some series [13, 17, 27]. It is also interesting to know that Brazilian isolates present a distinct clinical picture with immune manifestations (erythema multiforme), disseminated cutaneous lesions, and atypical forms [28, 29]. Such a varied disease spectrum has been attributed to factors like the mode of inoculation, the size and depth of the traumatic inoculum, the host immunity (fixed cutaneous sporotrichosis is considered to occur in patients with certain immunity against the fungus), and the virulence and thermotolerance of the fungus (the strains growing best at 35°C purportedly cause fixed cutaneous sporotrichosis and strains that grow both at 35°C and 37°C have been implicated for lymphocutaneous and extracutaneous disease) [12, 15, 30, 31]. However, the concept of thermosensitive strains of* S. schenckii* causing different clinical forms of the disease remains unsubstantiated [32]. Similarly, whether climate influences predominance of one or the other form also needs validation [33, 34].

### 3.1. Lymphocutaneous Sporotrichosis

**Figure 1:**
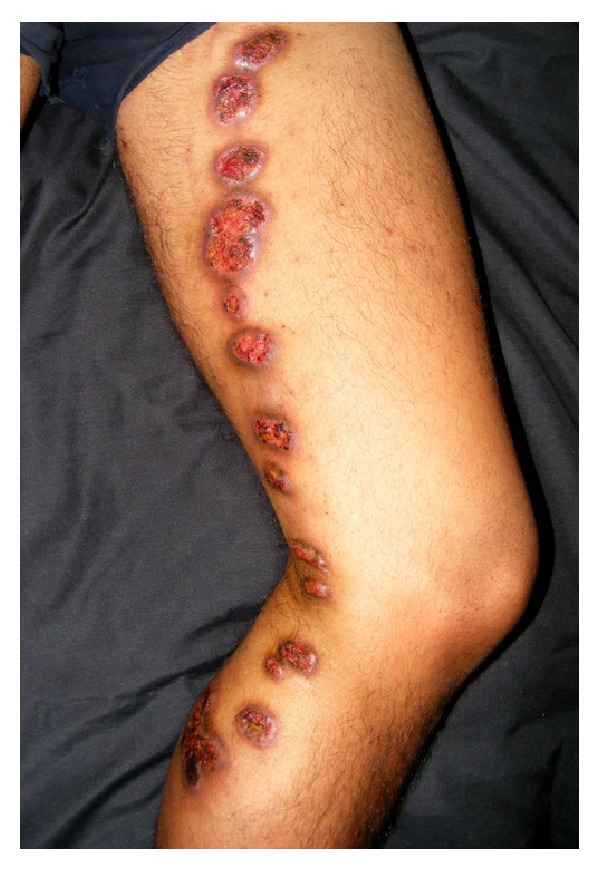
Lymphocutaneous sporotrichosis. Noduloulcerative lesions appear along the lymphatics proximal to the initial inoculation injury site.

It is the most common variety and accounts for 70–80% of the cases of cutaneous sporotrichosis [15, 35]. The extremities are affected most frequently. A noduloulcerative lesion (sporotrichotic chancre) at inoculation site and a string of similar nodules along the proximal lymphatics, with or without transient satellite adenopathy, characterizes this form (Figure 1). These secondary lesions appearing along lymphatics have varied morphology of erythematous papules, nodules, or plaques, having smooth or warty surface, and may soften and ulcerate discharging seropurulent material. They are mostly asymptomatic, may itch or become painful, and have indolent clinical course similar to that of the primary lesions.

### 3.2. Fixed Cutaneous Sporotrichosis

**Figure 2:**
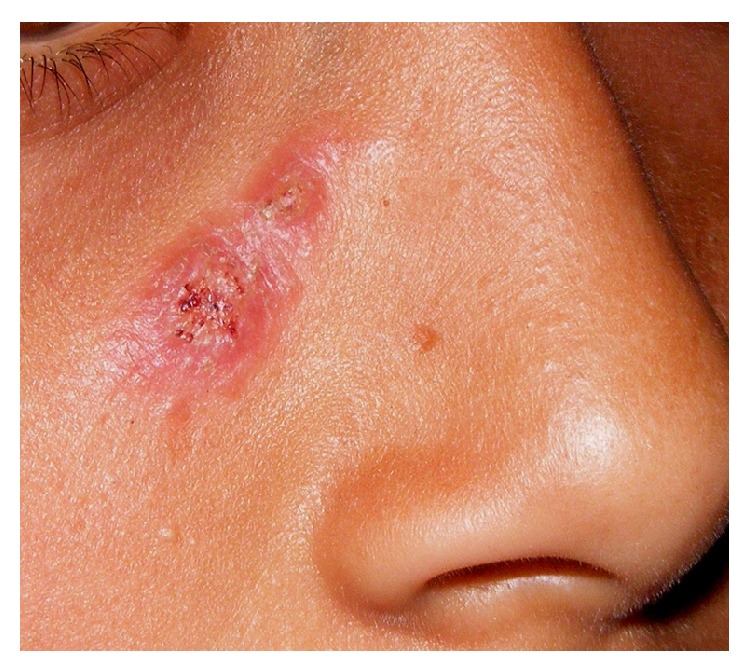
Fixed cutaneous sporotrichosis. A crusted/verrucous plaque develops at inoculation site, seen here over face of a child.

It occurs less commonly and is characterized by localized lesions at the inoculation site (Figure 2). Facial involvement occurs more frequently in fixed cutaneous sporotrichosis than in lymphocutaneous variety. The lesions are asymptomatic, erythematous, papules, papulopustules, nodules, or verrucous plaques and occasionally nonhealing ulcers or small abscesses. The lesions may resemble keratoacanthoma, facial cellulitis, pyoderma gangrenosum, prurigo nodularis, soft tissue sarcoma, basal cell carcinoma, erysipeloid, or rosacea [14, 36, 37]. This form is considered to occur usually among hosts having high resistance wherein minimal lesions may subside spontaneously or persist exceptionally if not treated, and responds better to treatment [15, 38].

### 3.3. Multifocal or Disseminated Cutaneous Sporotrichosis

This rarely described variety means ≥3 lesions involving 2 different anatomical sites implies cutaneous dissemination following multiple traumatic implantations of the fungus or rarely from hematogenous spread in individuals apparently having no predisposing factors for immunosuppression [15, 39, 40].

### 3.4. Extracutaneous Sporotrichosis

This form of the disease usually occurs in the setting of immunosuppression as from alcoholism, diabetes, AIDS, underlying malignancy, use of corticosteroids, or other immunosuppressive drugs [1, 41, 42]. Although sinusitis, pulmonary, ocular or central nervous system disease, meningitis, and endophthalmitis are the usual manifestations, osteoarticular sporotrichosis remains the most common systemic manifestation both in immunocompetent and in immunocompromised individuals that is often confused with other chronic inflammatory arthritis until destruction of adjacent bones or draining sinuses develop [14, 43–45]. Cutaneous lesions are uncommon in osteoarticular sporotrichosis and it usually begins as monoarticular disease without systemic illness. The pain is usually less severe than the bacterial arthritis but functional impairment may become severe in untreated cases. Sporotrichotic osteoarthritis usually affects knee, wrist, elbow, and ankle joints in order of frequency manifesting initially with tenosynovitis, joint effusion, bursitis, and synovial cyst formation [44]. Extensive destructive changes often occur in the affected joints because of delayed diagnosis that is very common owing to lack of clinical suspicion. Radiographs of involved joint usually show soft tissue swelling and osteoporosis of contiguous bones or show no abnormality. Parasynovial swelling, subchondral erosions, and narrowing of joint space are uncommon.

Pulmonary disease from inhalation of conidia is rare and characterized by cough, low-grade fever, weight loss, mediastinal lymphadenitis, cavitation mimicking tuberculosis, fibrosis, and rarely massive hemoptysis [46, 47]. Apical lesions resembling pulmonary tuberculosis may occur in 85% of these cases [1]. Most patients usually have underlying severe chronic obstructive pulmonary disease and may present with subacute/chronic pneumonia. Involvement of central nervous system/meningeal in immunocompromised patients usually has subtle changes in mental status as the only symptom.

## 4. Diagnosis

Clinical suspicion is the key for early diagnosis and cutaneous lesions need to be differentiated from cutaneous tuberculosis, cutaneous leishmaniasis, nocardiosis, chromoblastomycosis, blastomycosis, paracoccidioidomycosis, and atypical mycobacteriosis [15, 16, 48]. Ulcerating lesions can mimic pyoderma gangrenosum [49].

Direct smear examination of pus or biopsy specimen for causative fungus is not diagnostic because of paucity of fungal cells. Fine needle aspiration cytology from a lesion, particularly in extracutaneous or disseminated forms, may occasionally show epithelioid cell granuloma, asteroid bodies, and/or yeast cells and cigar shaped bodies when stained with periodic acid-Schiff (PAS) or Gomori-methenamine silver (GMS) stains [1, 14, 50]. Civila et al. [51] could demonstrate asteroid bodies of* S. schenckii* in almost 86% cases comprising 36 patients of lymphocutaneous and 6 patients of fixed cutaneous sporotrichosis; nearly 95% of these cases also yielded positive cultures. However, the sensitivity/specificity of direct microscopic examination of tissue sample remains understudied as most researchers consider it unhelpful diagnostic tool due to paucity of fungal cells. The culture of* S. schenckii* in artificial media remains gold standard in diagnosis. The animal inoculation studies are usually not needed for diagnosis.

### 4.1. Histopathology

Histopathology is usually nonspecific and mimics other granulomatous diseases (deep fungal infections, cutaneous tuberculosis, leprosy, sarcoidosis, and foreign body granulomas). The histologic features varies from acute on chronic inflammation with characteristic zonation to chronic epithelioid cell granuloma with foreign body or Langhans' giant cells at both ends and nonspecific chronic granulomatous inflammatory cell infiltration of the dermis in the middle of the spectrum [14]. The major histopathologic features of fixed cutaneous sporotrichosis include central ulceration of epidermis, hyperkeratosis at the edge, acanthosis, and epidermal hyperplasia that may vary to the extent of pseudoepitheliomatous hyperplasia. Neutrophilic abscesses may be seen in the dermis and/or epidermis. There is usually dense cellular infiltrate comprising lymphocytes plasma cells and variable number of epithelioid histiocytes, giant cells, and eosinophils (mixed granulomatous cellular infiltrate) in upper- and middermis with or without fibrocapillary proliferation [52, 53]. Nodules of lymphocutaneous sporotrichosis characteristically show 3 concentric zones; the central necrotic zone contains amorphous debris and polymorphonuclear leukocytes (zone of chronic suppuration); the middle tuberculoid zone is composed of epithelioid cells, giant cells (predominantly Langhans' type), and the outer zone comprising numerous plasma cells, lymphocytes, and fibroblasts with prominent capillary hyperplasia and proliferation (syphiloid zone) [14, 52]. However, this zonation becomes indistinct in older lesions. The fungal elements, when present, are visualized within these zones in PAS or GMS stained histologic sections. They appear globose, budding yeast-like cells measuring 3–8 *μ*m in diameter (in 84% cases), cigar shaped cells sized 1-2 × 4-5 *μ*m (in 33% cases) or oval to round or single budding forms of the yeast within the cytoplasm of giant cells or in the centre of asteroid bodies [1, 17, 54]. Asteroid bodies, sized 15–35 *μ*m in diameter, are usually seen in 40–85% of chronic sporotrichosis cases as extracellular and within the abscesses [1, 16, 17, 54]. Although observed more frequently in the lymphocutaneous variety, they do not differ morphologically from those seen in fixed cutaneous sporotrichosis. Their demonstration by direct immunofluorescence and specific immunohistochemical techniques is considered more sensitive and specific [55, 56]. Splendore-Hoeppli phenomenon, wherein one of the several fungal elements enveloped by an eosinophilic material radiating centrifugally in a sunburst fashion with its central portion reacting immunohistochemically with an anti-*Sporothrix schenckii* antibody, perhaps represents an immunologic interaction between the host and the pathogen [50–52]. However, the presence of asteroid bodies or Splendore-Hoeppli phenomenon is not pathognomonic and may be observed often in other granulomatous and/or infectious diseases as well; at best, they can aid in the diagnosis [51].

### 4.2. Culture and Identification of Causative Fungus

**Figure 3:**
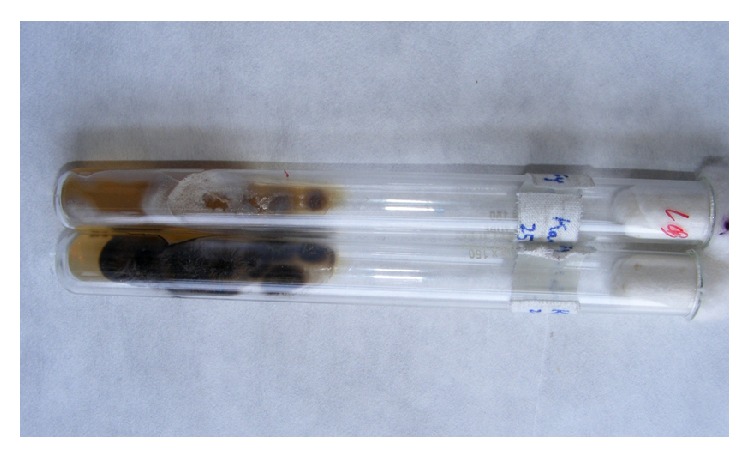
*Sporothrix schenckii* colony on Sabouraud's glucose agar (SDA) at 25°C. Initial cream color turns brown black as it matures.

The various* Sporothrix* species appear similar in morphology, but only* S. schenckii* is pathogenic to humans.* S. schenckii *can be grown from skin biopsy or other clinical samples (sputum, pus, synovial fluid/biopsy, bone drainage/biopsy, and cerebrospinal fluid) on Sabouraud's glucose agar (SDA), brain heart infusion agar, or Mycosel at 25°C and the growth is visible in 3–5 days to 2 weeks [1]. Incubation of cultures at 37°C in blood glucose-cysteine agar or brain-heart infusion broth will produce its yeast form. The initial cream-colored colonies grown on SDA at 25°C are smooth and moist but turn brown/black after a few weeks due to melanin production that protects it from phagocytosis and killing by human monocytes and macrophages and extracellular proteinases (Figure 3) [57].

**Figure 4:**
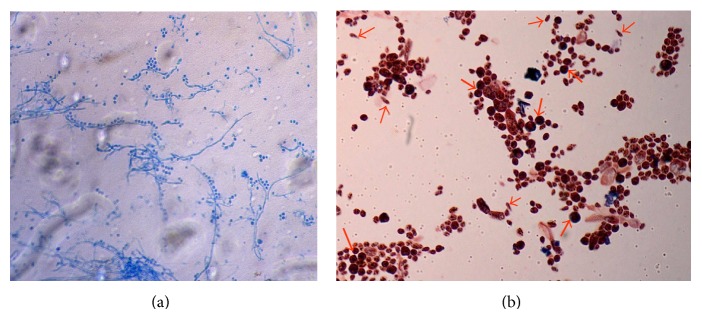
(a)* Sporothrix schenckii *from culture on SDA at 25°C. Seen here is delicate branching, mold form with pyriform conidia in characteristic flower-like arrangement or sleeve-like pattern (stain-lactophenol cotton blue ×40). (b) Yeast phase of* Sporothrix schenckii *isolate from culture on brain heart infusion agar at 37°C. Budding yeast cells (thick arrows) and cigar shaped yeast cells (thin arrows) interspersed between spores (Grams' stain, ×100) are seen here.

Microscopically, in lactophenol cotton blue mounts,* S. schenckii *appears as delicate branching septate hyphae with slender, short, conidiophores with tapering tips and surrounding pyriform conidia in a flower-like arrangement or as individual thick-walled, dark brown conidia attached directly to the hypha often in dense sleeve-like pattern (Figure 4(a)). In practice, S. schenckii is usually identified by its characteristic colony morphology, microscopic appearance and temperature dimorphism that is, its ability to exist as a mold at 25°C (room temperature) and as yeast at 37°C (in host tissues) (Figure 4(b)) [1, 58].

### 4.3. Intradermal Testing, Molecular and Other Diagnostic Techniques

The diagnostic value of all these tests is not reliable for diagnosis of sporotrichosis in view of significant variations in their specificity and sensitivity. Moreover, these tests remain to be of limited value in view of their unavailability for routine diagnostic use. Nevertheless, they are useful to raise a diagnostic suspicion prompting more aggressive diagnostic workup.

The diagnostic significance of intradermal tests to detect delayed hypersensitivity using sporotrichin or peptide-rhamnomannan (PRM) antigen remains ambiguous. They are negative in severe or disseminated sporotrichosis cases and are often positive in cured patients or in healthy people living in endemic areas [14, 15].

Analysis of antibody responses by immunoblotting and enzyme-linked immunosorbent assay (ELISA), agglutination, compliment-fixation tests, immunohistochemical, immunofluorescence, immunodiffusion, and immunoelectrophoresis techniques and detection of* S. schenckii* by polymerase chain reaction (PCR) or polymerase chain reaction-restriction fragment length polymorphism (PCR-RFLP) of calmodulin gene are useful in diagnosing rarer extracutaneous and disseminated forms [15, 59–61]. For instance, cerebrospinal fluid (CSF) antibody titers higher than serum antibody titers may be indicative of sporotrichotic meningitis. The ELISA has a reported sensitivity of 90% with 86% overall efficacy when tested against sera obtained from patients with any form of sporotrichosis [62]. Restriction fragment length polymorphism (RFLP) analysis of mitochondrial DNA is reportedly useful for identification, taxonomy, typing, and epidemiology of* S. Schenckii* [63]. Serological testing using antigenic fraction of the* S. schenckii* that binds concanavalin-A represents a more recent diagnostic tool [64].

### 4.4. Imaging Studies

Routine radio-imaging studies are usually not needed for the diagnosis of sporotrichosis unless specific systemic involvement is suspected. For instance, conventional X-rays or CT scan for the chest (pulmonary sporotrichosis) or other involved areas (osteoarticular sporotrichosis) will be supportive in the management but not specifically diagnostic of sporotrichosis.

## 5. Treatment Options

Spontaneous resolution is extremely rare and majority of the patients will require treatment. There are no well-controlled studies for the treatment of sporotrichosis and various treatment schedules recommended by Infectious Diseases Society of America are empirical and primarily based on case reports, retrospective reviews, and nonrandomized trials (Table 1) [65]. Low cost, ease of administration, safety profile, and the site of infection (localized or disseminated) often dictate the choice of therapy. Severe and systemic infection will require treatment that is more aggressive despite concern for drug-associated toxicities. Resolution of active infection and eradication of* S. Schenckii* from tissues remain the desired outcome of all treatments. The duration of 3–6 months for treatment of sporotrichosis remains arbitrary but any treatment must be continued for at least a period of 4 to 6 weeks after complete clinical remission to achieve mycological cure. Although treatment is prolonged and expensive, complete recovery without scarring is expected in cutaneous sporotrichosis following appropriate therapy. However, compromised pulmonary functions in pulmonary disease, severe disability from chronic osteoarticular sporotrichosis, or occasional scarring may result [36, 47, 66]. Patients with immunosuppression usually require life-long suppressive therapy.

Until recently and before the availability/approval of itraconazole, saturated solution of potassium iodide had been the standard treatment since the time it was introduced in 19th century and yet remains a first choice to treat uncomplicated disease in resource poor countries.

### 5.1. Saturated Solution of Potassium Iodide

Saturated solution of potassium iodide (SSKI) administered orally remains the low cost, first choice of the treatment for uncomplicated cutaneous sporotrichosis especially when high cost of itraconazole is precluding. However, it is not effective in extracutaneous form of sporotrichosis. The exact mechanism of its action against* S. Schenckii* remains poorly elucidated. Possibly, it inhibits granuloma formation through some immunologic and nonimmunologic mechanisms thereby exposing the fungus to the host defenses or other antifungal agents used concurrently [67]. However, it does not appear to increase monocyte or neutrophil killing of* S. Schenckii*. Interestingly,* S. Schenckii *can grow when plated with 10% SSKI suggesting that it has no fungistatic or fungicidal activity. It has been suggested that it gets converted to iodine* in*-*vivo* by myeloperoxidase, a hydrogen peroxide system of polymorphonuclear cells, and exerts its cidal effect as has been demonstrated from its inhibitory effect on germination of cells and their direct destruction on ultrastructure examination of* S. Schenckii *exposed to iodine-potassium-iodine solution [68].

SSKI is the most extensively used mode of treatment in both fixed cutaneous and lymphocutaneous sporotrichosis across countries especially from developing world where most cases occur and that too is without specific treatment trials. Failure of therapy unrelated to compliance is reported exceptionally [69, 70] and most reports delineate its efficacy to an extent that a favorable therapeutic response is considered nearly diagnostic in the absence of mycologic support [1, 13, 14, 17, 18, 39, 58, 71, 72]. It has been found effective even in cases not responding to itraconazole [73, 74]. However, no specific recommendations/guidelines or treatment schedules are available. SSKI, containing 1 g/mL of potassium iodide, is usually prescribed in a starting dose of 5 drops (using a standard eye dropper) three times a day (t.i.d.), taken orally mixed with fruit juice or milk to mask its unpalatable taste. The dose is increased daily by 5 drops t.i.d. up to a maximum dose of 30 to 40 drops t.i.d. until complete healing. The response becomes evident within 2 weeks and healing occurs in 4–32 weeks [14, 72]. However, this schedule remains incomprehensive particularly for patients working outdoors for long hours resulting in poor compliance. Cabezas et al. [75] compared the safety and efficacy of once-daily versus 3-times daily dosing in a randomized nonblinded clinical trial on 57 pediatric patients with culture confirmed fixed cutaneous or lymphocutaneous sporotrichosis. The starting dose of SSKI 150 mg/day was increased to maximum of 160 mg/day in both groups. Although, adverse events were higher (61% versus 42%) with once-daily dose, the cure rates were comparable in both groups.

Adverse effects such as metallic taste, flu-like syndrome, excessive lacrimation, gastrointestinal upsets, parotid swelling, acneiform or papulopustular eruptions, exacerbation of dermatitis herpetiformis, and lesional pain and inflammation in rare instances may lead to noncompliance in as high as 60% cases but rarely need discontinuation of treatment [13, 15, 17, 39]. Metallic taste signifies threshold of maximum tolerable dose and the dosage is adjusted at a lower level. Hypothyroidism or hyperthyroidism, iododerma, cardiac irritability, vasculitis, pustular psoriasis, pulmonary edema, urticaria and angioedema, myalgia, lymphadenopathy, and eosinophilia are some potential adverse reactions [14, 76]. Hypothyroidism associated with SSKI therapy is usually precipitated in patients already having defective (partial) autoregulation mechanism (as from Hashimoto's thyroiditis, surgery, or radioactive iodide therapy for Graves' disease) that maintains thyroid hormone synthesis. Thyroid gland stops producing thyroid hormone by negative feedback mechanism when excess quantities of iodine exist (Wolff-Chaikoff effect). Inbuilt autoregulation mechanism maintains a storage pool of organic iodine in the thyroid gland and ensures that it produces enough thyroid hormone for the patient to remain euthyroid (escape phenomenon) [76]. In the complete absence of autoregulation mechanism (as in patients from areas of iodine deficiency having long-standing goiter) due to presence of autonomous thyroid foci the thyroid synthesizes excess thyroid hormone leading to thyrotoxicosis (Jod-Basedow disease). However, unless preexisting thyroid disease is suspected baseline thyroid function studies are not required as the therapeutic effect usually occurs in few weeks and within the period of “escape phenomenon.” Discontinuation of SSKI will usually restore normal thyroid functioning within a month in case of iatrogenic hypothyroidism. Patients taking angiotensin-converting enzyme inhibitors, potassium-sparing diuretics, or with renal impairment may develop potassium toxicity and need careful monitoring during SSKI therapy. SSKI is currently pregnancy category D drug. Therapy with SSKI should not be reinstituted in patients developing “flu-like syndrome”/hypersensitivity to SSKI as they will suffer adverse reactions even at low doses.

### 5.2. Azoles (Itraconazole, Fluconazole, and Ketoconazole)

All azoles inhibit cytochrome P450 enzyme system and concomitant administration of drugs metabolized by this enzyme system (digoxin, warfarin, phenytoin, carbamazepine, phenobarbitone, rifampicin, cisapride, astemizole, triazolam, midazolam, lovastatin/simvastatin, H-2 antagonists, and oral hypoglycemic agents) remains contraindicated because of potential serious toxicities. They are also contraindicated in pregnancy and hypersensitivity to azoles.

*Itraconazole*, the oral antifungal agent from azoles, in a dose of 100–200 mg daily is effective and well-tolerated and has largely replaced SSKI and amphotericin B with its 90–100% efficacy rates in cutaneous as well as extracutaneous sporotrichosis [77]. It inhibits enzyme cytochrome P450 lanosterol 14*α*-demethylase that converts lanosterol to ergosterol, the main sterol in fungal cell wall. Its minimum inhibitory concentration (MIC) of 0.1–1.0 mg/L for the yeast form is well achieved with the recommended therapeutic doses and has concentration in stratum corneum that is nearly 10-fold higher than the plasma levels. Itraconazole is also used in pulse regimens as it persists in the stratum corneum for 3-4 weeks (for 6–12 months in nails) after discontinuation. Despite its high cost it has become the drug of choice for treating both cutaneous (fixed and lymphocutaneous) and osteoarticular varieties of sporotrichosis with success rates varying between 90 and 100% (60%–80% for osteoarticular sporotrichosis) [60, 75]. It is administered orally in a dose of 200–400 mg/day (minimum dose 200 mg/day) for a period of 3 to 6 months (1 year for osteoarticular and disseminated forms) as per current guidelines from Infectious Diseases Society of America (IDSA) [65]. Adequate response without recurrences or adverse effects has been observed in both fixed and lymphocutaneous sporotrichosis even at 100 mg daily given for a mean duration of 18 weeks but higher doses between 150 and 200 mg daily are generally favored [26, 78, 79]. It is an acceptable alternative in patients who are intolerant to SSKI or when itraconazole is easily available/affordable despite reported relapses or therapeutic failure [73, 74, 79]. Fixed cutaneous variety responds at lesser duration than lymphocutaneous form. However, higher doses are generally recommended for poor responders or relapsed cases but are associated with adverse drug reactions [80]. Bonifaz et al. [81] by using oral itraconazole 400 mg/day for one week with a 3-week break (pulse therapy) achieved clinical and mycological cure in their 4 of 5 patients with cutaneous sporotrichosis (one of four patient with lymphocutaneous variety was lost to follow-up, and one patient had fixed cutaneous sporotrichosis). Thereafter, the drug was administered as pulses until clinical and mycological cure that was achieved after 2 (for fixed cutaneous variety) to 5 pulses. Song et al. [82] further compared oral itraconazole pulse therapy (200 mg b.i.d. for 1 week and off for 3 weeks, average pulses 2.65 ± 0.81) with daily dosing (100 mg b.i.d., mean duration 2.80 ± 2.33 months) in prospective, randomized, evaluator blinded study comprising 25 patients with cutaneous sporotrichosis in each group. Although more patients receiving continuous therapy showed cure (95.8%) than those receiving pulse therapy (81.8%) at 48 weeks, the results were not statistically significant. However, not many studies are available on this mode of treatment for making any recommendation. It was moderately effective in osteoarticular sporotrichosis with a cure rate of 73% in a small series of 11 patients who had relapsed after initial cure [75]. However, all 6 patients of osteoarticular sporotrichosis responded to itraconazole in another study [83]. Nevertheless, relapses have occurred 1–7 months after treatment duration of 6–18 months or even after repeated treatment with itraconazole [79]. Nausea and epigastric pain, hypercholesterolemia or hypertriglyceridemia, altered liver function tests (rarely serious hepatotoxicity), headache, and peripheral edema are some of its reported adverse effects observed more frequently with higher doses. Variable therapeutic outcome is another limitation for its use. No dosage adjustment is usually needed in patients with hepatorenal dysfunction.

*Fluconazole* is a synthetic broad-spectrum bistriazole antifungal agent that selectively inhibits fungal cytochrome P-450 that is necessary for sterol C-14 alpha-demethylation to ergosterol, an essential for fungal cytoplasmic membrane integrity. This leads to abnormal permeability, membrane bound enzyme activity, and coordination of chitin synthesis. Used orally alone or in combination with SSKI, fluconazole provides another useful therapeutic option. It has been used to treat both fixed and lymphocutaneous sporotrichosis in doses between 150 mg once a week and 200 mg daily [84, 85]. However, higher doses between 400 and 600 mg/day are generally recommended especially for treating visceral and osteoarticular sporotrichosis but the response generally remains poor [36, 72, 86, 87]. Prolonged therapy with doses of 400–800 mg/day given for several months has been associated with alopecia [86]. Nausea, vomiting and diarrhea, abdomen pain, headache, and abnormal liver enzymes observed commonly rarely necessitate discontinuation of treatment. It is contraindicated in pregnancy because of its teratogenic potential [88]. As the experience with fluconazole therapy in sporotrichosis is limited, it remains a second-line treatment option for patients intolerant to itraconazole for being moderately effective.

Response to* Ketoconazole* remains discouraging and it is not recommended to treat sporotrichosis [14, 79, 89]. Sharkey-Mathis et al. [79] observed complete failure of treatment with ketoconazole at doses as high as 400–600 mg given daily for 7–9 months. Apart from low efficacy, even lower than fluconazole, hepatotoxicity is another limitation for its use.

### 5.3. Allylamines (Terbinafine)

Terbinafine is another alternative agent to treat cutaneous or lymphocutaneous sporotrichosis that is unresponsive to itraconazole or when itraconazole is not tolerated. It is well absorbed following oral administration, has low binding to microsomal cytochrome P450 enzyme, and has no effect on bioavailability of other drugs metabolized by this enzyme system. Being highly lipophilic its antifungal activity is maintained from adipose tissue depots few weeks after discontinuation of treatment. It inhibits ergosterol synthesis by inhibiting squalene epoxidase for exerting its fungicidal effect. It is effective against most dermatophytes,* Aspergillus* species, blastomycosis, histoplasmosis, and* Scopulariopsis brevicaulis* and other fungi including* S. schenckii.* It has a long half-life and has been shown to have good* in vitro* activity against* S. schenckii* (MIC range of 0.007–0.5 *μ*g/mL) [90]. However, there is no consensus for optimal dosage and duration schedule for terbinafine. It has been used alone or in combination with SSKI to treat few patients in doses ranging from 125 to 1000 mg/day given for 4–37 weeks to achieve clinical cure in small series or case reports [90, 91]. Francesconi et al. [90] achieved cure with terbinafine 250 mg daily without recurrences in 96% of 50 patients with cutaneous sporotrichosis being on simultaneous treatment for other comorbidities like hypertension, diabetes mellitus, dyslipidemias, arrhythmia, and so forth. Its efficacy in a dose of 250 mg/day is reportedly comparable with itraconazole 100 mg/day (92.7% versus 92%) administered for mean duration of 11.5 and 11.8 weeks, respectively [92]. However, terbinafine regimens using higher doses have shown superior efficacy. Terbinafine 500 mg b.i.d. administered for a mean of 13.9 ± 6.7 weeks cured 87% of 35 patients as compared to 52% of 28 patients receiving 250 mg b.i.d. for a mean of 17.7 ± 5.8 weeks in a multicenter, double blind, randomized clinical trial [93]. It is currently a pregnancy category B drug.

### 5.4. Polyenes (Amphotericin B)

Amphotericin B (deoxycholate), a lipophilic polyene macrolide antibiotics synthesized from* Streptomyces nodosus*, is available mainly for intravenous or topical use as an antifungal agent. It binds to ergosterol or other sterols in the fungal cytoplasmic membrane causing mechanical interruption enhancing permeability of fungal cell membrane for monovalent ions (sodium, potassium) and other molecules and cell death. However, its main fungicidal activity has been attributed to its ability to cause autooxidation of the cytoplasmic membrane and release of lethal free radicals. It is safe during pregnancy and is a drug of choice for treating severely recalcitrant and disseminated cutaneous and/or pulmonary/meningeal sporotrichosis or HIV-associated disease. It may also be indicated in patients with extensive osteoarticular sporotrichosis or who are not responding to adequate itraconazole therapy but its intra-articular use is considered unwarranted [75]. After initial 1 mg intravenous testing dose, the recommended adult dose of 0.25 mg/kg/day is increased to achieve the targeted dose of 2-3 gm [26]. Fever, chills, headache, malaise, and vomiting that may occur during drug administration can be ameliorated by premedication with sedatives, antipyretics, and corticosteroids. Hypokalemia, hypomagnesemia, nephrotoxicity, and reversible normochromic normocytic anemia are common adverse effect and require regular once-weekly monitoring. Cardiovascular and bone marrow toxicity occurs rarely [26]. Availability of new lipid formulations of amphotericin B has improved its safety profile by delivering higher concentrations of the drug and decreased nephrotoxicity. It is usually used in a dose of 3–5 mg/kg of lipid formulation or 0.7 to 1.0 mg/kg/day of amphotericin B deoxycholate depending upon the severity of the disease [65]. Although there are no guidelines in view of variable therapeutic response from amphotericin B, it is usually recommended in the initial phase of therapy, until a favorable response is achieved, followed by itraconazole for rest of the treatment course [65].

### 5.5. Flucytosine

This synthetic fluorinated pyrimidine acts synergistically with amphotericin B to reduce its dose to lessen its toxicity. Amphotericin B enhances permeability of fungal cell wall and facilitates penetration of flucytosine. Its deamination occurs within fungal cells to fluorouracil that disrupts fungal RNA and DNA metabolism. The usual dose is 50–150 mg/kg/day given four times daily for 6–12 weeks [26]. Cure has been achieved for disseminated sporotrichosis of skin and bone with a combination of amphotericin B (4.8 gm) and 5-fluorocytosine (8 gm/day, 100 mg/kg/day) in 6 months [94]. However, its use is no longer recommended due to serious adverse reactions (gastrointestinal intolerance, bone marrow toxicity, and photosensitivity) and availability of better alternatives.

### 5.6. Newer Antifungals

The use of posaconazole or ravuconazole in patients with sporotrichosis remains understudied. Posaconazole has shown good activity against all the five species of* S. schenckii* complex and activity of ravuconazole is limited to only against* S. brasiliensis* in* in vitro *studies [83]. Echinocandins has shown no activity against* S. schenckii* and voriconazole is not recommended for treatment of sporotrichosis due to its limited* in vitro* activity against* Sporothrix *spp. [95–97].

### 5.7. Antifungal Susceptibility Profile

The variability in therapeutic efficacy and* in vitro* activity demonstrated in different studies is attributed to the fact that* S. schenckii* is a complex of different species. For instance, itraconazole has shown good* in vitro* activity against* S. brasiliensis* whereas the drug showed high minimum inhibitory concentrations when tested for the other species [95]. Terbinafine was the most active drug in* in vitro* studies as compared to itraconazole, ketoconazole, or posaconazole against human and animal isolates of* S. schenckii* while* S. brasiliensis* showed the best response to antifungals and* S. mexicana* had the worst response [95, 98].* S. globosa* and* S. schenckii* were found to be itraconazole-resistant strains while terbinafine was the most active drug, followed by ketoconazole and itraconazole, and fluconazole and voriconazole were less effective in a recent* in vitro* study from Brazil [99]. In a similar study from Sao Paulo (Brazil), itraconazole and posaconazole were moderately effective against* S. schenckii* and* S. brasiliensis* while flucytosine, caspofungin, and fluconazole showed no* in vitro* antifungal activity against any of* Sporothrix* species (*S. schenckii*,* S. brasiliensis*, and* S. mexicana*) in another study [100]. Posaconazole has shown efficacy for* S. brasiliensis* and* S. schenckii *sensu stricto infections in experimental murine models [101]. Voriconazole had not shown antifungal activity against* S. brasiliensis* and had only fungistatic effect in mice infected with* S. schenckii *sensu stricto [102].

### 5.8. Thermotherapy

Temperatures above 38.5°C are detrimental to the growth of* Sporothrix* and directly damage the pathogen and, in addition, local hyperthermia is considered to enhance intracellular killing capability of neutrophils [103]. The daily application of local heat (42°-43°C) to the lesion for weeks using heat compresses or hand held pocket warmer, infrared or far infrared heater is recommended for treating small lesions in fixed cutaneous variety or cutaneous sporotrichosis in pregnant women pending specific therapy. Its reported cure rate is 71% among 14 patients in a study by using different modes of thermotherapy but in general its efficacy remains largely unevaluated [65, 71, 104]. Nonetheless, it can best be combined with SSKI or itraconazole for adjunctive benefit. The heat is usually applied for 15–60 minutes several times daily until the lesions heal or specific therapy is instituted [71].

### 5.9. Surgical Interventions

Surgical excision is usually not recommended, as it is not unusual for the disease to get destabilized and disseminate following minor trauma such as biopsy procedure [36]. However, invasive procedures are required for obtaining clinical samples such as full-thickness skin or bone biopsy, arthrocentesis for synovial tissue biopsy, or bronchoscopy with bronchoalveolar lavage for culture and transbronchial biopsy to establish laboratory diagnosis. Similarly, surgical therapy sometimes becomes unavoidable as in the management of osteoarticular sporotrichosis along with antimicrobial therapy for eradication of infection as for any other bone and joint infections. Joint damage can be minimized by appropriate drainage of infected joints or debridement of sequestrum but arthrodesis may be needed when joint destruction ensues [66]. Combination of surgical resection of the involved lung segment and amphotericin B is considered better than either mode used alone for pulmonary sporotrichosis [65]. Nevertheless, surgical excision/debridement as a sole therapy is neither effective nor recommended and it will always be prudent to combine surgical procedures with appropriate drug therapy for better therapeutic outcome.

### 5.10. Treatment of Sporotrichosis in Pregnant Women and Children

SSKI is currently pregnancy category D drug and remains contraindicated in pregnant/lactating women for possible development of neonatal hypothyroidism and/or thyromegaly unless benefits outweigh the risks. Terbinafine is not approved for use in pregnancy and azoles must be avoided. Local hyperthermia can be used to treat cutaneous sporotrichosis that does not require urgent therapy. However, for sporotrichosis that must be treated during pregnancy, liposomal amphotericin B (3–5 mg/kg/day) can be used. As there is no risk of worsening of sporotrichosis or its dissemination to the fetus, treatment is best delayed in pregnant patients. Cases of sporotrichosis in children have been reported less frequently than in adults. Nevertheless, they are not uncommon. Pediatric patients with sporotrichosis can be treated with an equivalent to half the adult dose of SSKI, up to a maximum of 15 drops thrice daily or itraconazole 6–10 mg/kg/day (max 400 mg/d) for 3-4 months as in adults [14, 27]. Most clinicians prefer itraconazole as their first choice of treatment in children for convenient once a day dosing. SSKI, itraconazole, and terbinafine have been used successfully to treat sporotrichosis in infants aged <10 months [105]. However, availability of pediatric formulations of antifungal agents remains a limitation. Local heat therapy is another useful option for limited cutaneous disease in children.

### 5.11. Treatment of Sporotrichosis in Patients with Immunosuppression or HIV Disease

Predisposing conditions responsible for immunosuppression like diabetes mellitus, chronic alcoholism, myeloproliferative disorders, immunosuppressive therapy for organ transplant, autoimmune disorders, or cancers, prolonged treatment with systemic corticosteroids and HIV infection have been implicated for extracutaneous sporotrichosis, an opportunistic form of infection. Cutaneous lesions are usually multiple and widespread. Cutaneous dissemination with or without systemic involvement can occur in patients with AIDS. Extracutaneous sporotrichosis in the form of invasive sinusitis, ocular sporotrichosis, pulmonary infection, osteoarthritis, and spread to central nervous system has been documented [2, 3, 106]. Immunocompromised patients having dissemination of infection to central nervous system/meninges usually manifest with subtle changes in mental status as the only symptom. Liposomal amphotericin B in recommended doses is the drug of first choice in these patients and they will need suppressive therapy with oral itraconazole 200 mg/d for life after the clinical cure, as eradication of the pathogen may not be possible at all.

## 6. Comments

Cutaneous and subcutaneous involvement in sporotrichosis is more common than disseminated forms and can be treated readily, if the cost is not precluding, with oral itraconazole 200 mg/d for 2–4 weeks after all lesions have healed usually for 3–6 months. Patients not responding to this regimen should get either oral itraconazole 200 mg twice daily, oral terbinafine 500 mg twice daily, or SSKI in standard dosing schedule. The osteoarticular, visceral, and disseminated forms of sporotrichosis are uncommon and occur more often in patients with immunosuppression and are difficult to treat. Oral itraconazole 200 mg twice daily for at least 12 months is generally recommended for osteoarticular sporotrichosis and less-severe disease. Liposomal amphotericin B (3–5 mg/kg/day) can be used for initial therapy with subsequent switch to oral itraconazole for severe or life-threatening pulmonary sporotrichosis, meningeal sporotrichosis, and disseminated sporotrichosis. Upon clinical stabilization, therapy can be changed to oral itraconazole 200 mg twice daily for a minimum of 12 months.

Recent concept that* S. schenckii* is a complex of different phylogenic species with different geographic distribution, virulence, and* in vitro* susceptibility to antifungal agents has helped in understanding variability of therapeutic response observed across antimicrobial agents. This perhaps will form the basis for future research particularly in therapeutics that remains under studied and lacks well-designed controlled clinical trials. The possibility of yeast form of* S. schenckii* being more susceptible to various antifungal agents also needs to be explored further [107]. Infectious Diseases Society of America currently recommends itraconazole as first line treatment for subcutaneous sporotrichosis. However, SSKI still forms the preferred treatment in most developing countries where major chunk of the disease occurs. Thus, improved formulations of SSKI that are more palatable, have simplified administration regimen, and have improved compliance are highly desirable. The observed improved efficacy of itraconazole in combination with SSKI versus either drug used alone in phaeohyphomycosis requires evaluation in the treatment of sporotrichosis as well [108]. The possible efficacy of topical eberconazole and topical formulations of amphotericin B in cutaneous sporotrichosis, as in case of cutaneous leishmaniasis, too needs assessment [107, 109, 110]. There also remains a scope for new therapies that can cure the disease in shorter period of time and treat the disseminated forms effectively. Inhibition of melanin formation that protects the fungus from body's immune system can perhaps be another target for development of new therapeutic agents and requires further research. As early diagnosis remains a challenge for the treating physicians, development of new diagnostic tools with shorter turnaround time than culture is another area for future researchers. Patient education for preventive aspects for minimizing the risk and counseling for protracted, prolonged treatment will benefit in the long-term.

## Figures and Tables

**Table 1:** Recommendations by Infectious Diseases Society of America for Sporotrichosis Treatment^*^.

Sr. number	Clinical manifestations	Preferred treatment [dose]	Alternative treatment	Remarks
1	Uncomplicated cutaneous sporotrichosis	Itraconazole [200 mg/day]	Itraconazole [200 mg b.i.d.] or terbinafine [500 mg b.i.d.] or SSKI [increasing doses] or fluconazole [400–800 mg/day] or local hyperthermia	Treatment for 2–4 weeks after lesions have resolved

2	Osteoarticular sporotrichosis	Itraconazole [200 mg twice daily (b.i.d.)]	Liposomal amphotericin B (3–5 mg/kg/day) or deoxycholate amphotericin B [0.7–1 mg/kg/day] until resolution	Switching to itraconazole after resolution and treatment for a total of 12 months

3	Pulmonary sporotrichosis	Liposomal amphotericin B [3–5 mg/kg/day] and then itraconazole [200 mg b.i.d.]	Deoxycholate amphotericin B [0.7–1 mg/kg/day] until recovery and then itraconazole [200 mg b.i.d.]	Treating less severe disease with itraconazole. Treatment for at least 12 months

4	Meningeal sporotrichosis	Liposomal Amphotericin B [3–5 mg/kg/day] and then itraconazole [200 mg b.i.d.]	Deoxycholate amphotericin B [0.7–1 mg/kg/day] until recovery and then itraconazole [200 mg b.i.d.]	Length of therapy with amphotericin B is not established. Treatment for 4–6 weeks and total of 12 months. Suppressive therapy with itraconazole is needed

5	Disseminated sporotrichosis	Liposomal amphotericin B [3–5 mg/kg/day] and then itraconazole [200 mg b.i.d.]	Deoxycholate amphotericin B [0.7–1 mg/kg/day] until recovery and then Itraconazole [200 mg b.i.d.]	Treatment with amphotericin B until objective improvement and for at least 12 months. Suppressive therapy with itraconazole is needed

6	Sporotrichosis in pregnant women	Treating only severe sporotrichosis with liposomal amphotericin B [3–5 mg/kg/day] or deoxycholate amphotericin B [0.7–1 mg/kg/day]. Treatment with local hyperthermia [approx. 45°C] for uncomplicated cutaneous sporotrichosis		Preferably, defer treatment for uncomplicated cases

7	Sporotrichosis in children	Itraconazole [6–10 mg/kg/d or maximum of 400 mg/day] for mild disease, deoxycholate amphotericin B [0.7–1 mg/kg/day] for severe disease	SSKI with increasing doses equivalent to half the adult dose for a duration as in adults	Treating severe disease with an amphotericin B formulation

^*^Modified after Kauffman et al. [65].
